# Beta-Blocker Use and Risk of Hip Arthroplasty in Osteoarthritis: A Retrospective Electronic Health Record Study

**DOI:** 10.3390/life15081326

**Published:** 2025-08-20

**Authors:** Ping-Hao Chiang, Yang-Chi Lin, Jing-Yang Huang, Yun-Che Wu

**Affiliations:** 1 Department of Medical Education, Chiayi Chang Gung Memorial Hospital, Chiayi 613016, Taiwan; bryan880902@cgmh.org.tw; 2 Department of Medical Education, Chung Shan Medical University Hospital, Taichung 40201, Taiwan; cshy2247@csh.org.tw; 3 Department of Medical Research, Chung Shan Medical University Hospital, Taichung 40201, Taiwan; cshe961@csh.org.tw; 4 Department of Orthopedics, Taichung Veterans General Hospital, No. 1650, Section 4, Taiwan Boulevard, Xitun District, Taichung 40705, Taiwan

**Keywords:** osteoarthritis of the hip, total hip arthroplasty, beta-blockers, selective beta-blocker, non selective beta-blockers, cohort study

## Abstract

Objectives: This study aimed to determine whether prior use of a beta blocker (BB) is associated with the three-year risk of total hip arthroplasty after being diagnosed with osteoarthritis of the hip and compare risks across BB subtypes. Methods: Through the TriNetX database, patients with hip OA were included and further divided into the with- and without-BB cohorts. BB users were defined as patients with prescriptions between 1 day and 1 year before hip OA diagnosis and at least one refill afterward. The index date was defined as the diagnosis date of hip OA. Moreover, the BB cohorts were split to evaluate the difference between different types of beta-blocking agents. After propensity score matching, a three-year risk of undergoing THA was calculated. Results: A total of 313,430 patients were involved in this study, including 23,580 with BB usage, and 289,850 without. After propensity score matching, 23,096 patients remained in each cohort. For the with- and without-BB cohort, the average ages were 69 ± 11.9 and 63.3 ± 11.4, with a majority being female (52.4% vs. 56%). After the three-year follow-up, the use of BBs (2333 vs. 1539, HR = 1.494; 95% C.I. = 1.4–1.593) was associated with a higher risk of undergoing THA. Furthermore, among the three types of BBs, the use of alpha-BBs showed the highest hazard ratio when compared to the without-BB cohort (788 vs. 470, HR = 1.639; 95% C.I. = 1.462–1.837). Conclusions: These findings suggest a potential association between BB use and hip arthroplasty in OA patients, warranting further investigation rather than immediate changes in clinical practice. Exploration into the detailed mechanisms is warranted and merits investigation in future studies.

## 1. Introduction

Osteoarthritis (OA) is currently the most common arthritis worldwide. With the global aging population and the prevalence of obesity [1], the social cost associated with OA is steadily rising. Moreover, OA is a leading cause of disability among the elderly [2]. Speaking of the treatment of OA, aside from non-pharmacological interventions, oral nonsteroidal anti-inflammatory drugs (NSAIDs) are the most common pharmacological treatment. Glucocorticoid injections, and drugs like duloxetine and opioids, also play roles in management [3]. However, despite the lack of strong surgical indications in hip OA patients, total hip arthroplasty (THA) is indicated in cases of severe pain, functional impairment, abnormal imaging findings, or when pharmacological treatment fails to meet patient expectations [4]. Some patients with poor functional status or advanced age may decline surgery after individualized risk–benefit assessment. Even though the evolving landscape of surgical options [5,6,7], THA remains a crucial benchmark for poor outcomes in hip OA. Due to the burdens imposed by the surgical process and postoperative care on individual patients and society, favorable or adverse factors influencing the risk of THA for hip OA were widely discussed.

Previous studies have indicated an association between the use of beta blockers (BBs) and a lower risk of OA. This may stem from the potential role of BBs in regulating pain [8]. Consequently, research has focused on whether BBs can improve pain conditions in OA patients and potentially reduce the use of opioid analgesics [9]. The cardiovascular system regulated by BBs is also crucial for coupling osteogenesis, thus supporting bone homeostasis and repair [10]. Additionally, both the joints and the cardiovascular system are influenced by immune responses, exhibiting interrelated effects [11]. However, regarding total joint replacement, which is considered a last-resort treatment, there is currently a lack of evidence confirming the association between BBs and their risks. Even after the emergence of a nested case–control study, it only tentatively suggests the potential reduction in the risk of undergoing total knee arthroplasty (TKA) following knee OA [12]. Although prior studies have reported that β-blockers (BBs) may attenuate pain or delay joint replacement in knee OA, the extrapolation of such findings to hip OA remains speculative. This is not only due to anatomical and biomechanical differences—such as the hip joint’s deeper articulation, different loading mechanics, and distinct vascular supply—but also because the sympathetic innervation patterns vary significantly between the two joints. These factors may influence how BBs interact with nociceptive pathways and subchondral remodeling in the hip. Therefore, bridging preclinical findings with population-level outcomes requires careful consideration of these joint-specific nuances.

Interestingly, due to the diversity of drugs, some BBs also have the function of blocking alpha-adrenergic receptors [13,14]. In addition, they are also classified into selective and non-selective beta-adrenergic receptor blockers [15]. Through statistical analysis of patients using these drugs, insights into the impact of adrenergic receptors on joints may be gleaned.

Beyond their cardiovascular effects, BBs modulate sympathetic nervous system activity, which in turn regulates subchondral bone remodeling, cartilage metabolism, and local vascular tone [16,17]. Alpha–BBs inhibit peripheral alpha-adrenergic receptors, leading to systemic vasodilation. In the context of the hip joint, this may impair microvascular perfusion, potentially accelerating cartilage degradation and bone turnover, thereby increasing the likelihood of requiring surgical intervention such as total hip arthroplasty.

In this context, we hypothesize that BB use is associated with differential risks of hip arthroplasty among patients with hip OA, and that such risks may vary across BB subclasses (e.g., selective β1-blockers, non-selective BBs, and α–β blockers), each with distinct receptor profiles and hemodynamic effects [18]. This study aims to investigate 1. whether patients using BBs at the time of hip OA diagnosis is associated with the risk of THA compared to those who have never taken BBs; 2. whether there are differences in THA risk between taking alpha-beta adrenergic receptor blockers (aBBs), non-selective beta-adrenergic receptor blockers (non-selective BBs), and selective beta-1 adrenergic receptor blockers (selective BBs) when hip OA diagnosed; and 3. the impact of BBs on THA hazard at different time intervals and in different populations.

## 2. Methods

### 2.1. Data Collection

The data used in this study was collected on 25 June 2024, from the TriNetX [19,20] US Collaborative Network, which provides access to electronic health records of osteoarthritis of the hip, congenital deformities of the hip, osteonecrosis, total hip arthroplasty, and the beta-blocking agent from approximately 104 million patients in approximately 60 healthcare organizations. This retrospective study is exempt from informed consent. The data reviewed was from a secondary analysis of existing data, did not involve intervention or interaction with human subjects, and was de-identified per the de-identification standard defined in Section §164.514 (a) of the HIPAA Privacy Rule. The process of de-identifying data is attested to through a formal determination by a qualified expert as defined in Section §164.514 (b) (1) of the HIPAA Privacy Rule. This formal determination by a qualified expert was refreshed in December 2020. In addition, patients were not involved in the design, conduct, reporting, or dissemination plans of our research.

### 2.2. Population

**Figure 1:**
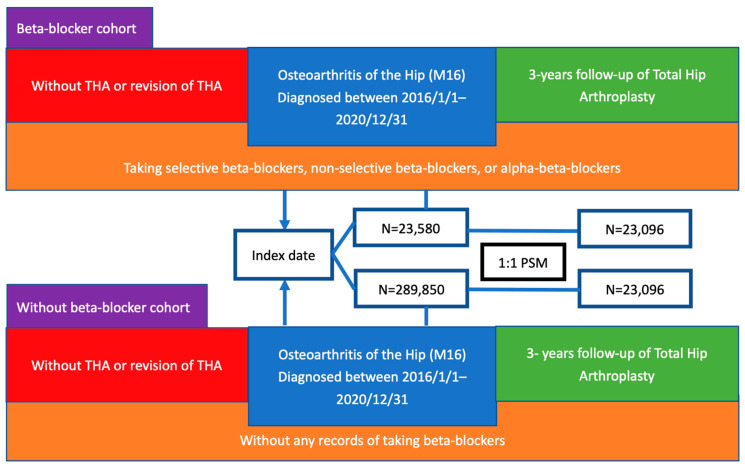
Flowchart.

This study focused on patients with hip OA. The inclusion criteria comprised all patients whose hip OA was diagnosed between 1 January 2016 and 31 December 2020. Patients with congenital deformities of the hip or osteonecrosis were excluded because these conditions represent distinct pathological entities with different surgical indications and progression trajectories, which could confound the association between BB use and THA risk in primary OA. Furthermore, patients with any prior THA-associated procedure records were also excluded to avoid possible reporting bias. Diagnosis and treatment were conducted using electronic codes, and detailed coding was provided in Supplementary Table S2. In this study, the diagnosis date of hip OA was considered the index date for the analysis. After that, patients were further divided into the BB cohort and the non-BB cohort. To define the BB cohort, we included only patients with prescription records for BB between 1 day and 1 year before the index date (hip OA diagnosis) and at least one documented refill after the diagnosis to prevent possible misclassification bias. This criterion was selected to ensure continued use rather than incidental or one-time prescriptions. However, due to limitations in dosage granularity within TriNetX, we did not impose a minimum dose threshold. The non-BB cohort was defined as individuals who had never used BBs (Figure 1). In the analysis, BBs were classified into selective beta-1 blockers (selective BBs), non-selective beta-blockers (non-selective BBs), and combined alpha- and beta-blockers (aBBs).

### 2.3. Propensity Score Matching

In the analyses, the cohorts underwent propensity matching (1:1) for the confounding factors. First, cohorts were categorized by age at index, sex, and ethnic background (White Americans, Black or African Americans, Asian Americans, and others) before commencing the statistical evaluation of outcome risks. Other known covariates or plausible associations with either the progression of hip osteoarthritis or the likelihood of undergoing THA were included. For instance, comorbidities such as hypertension, diabetes, and chronic kidney disease can influence surgical candidacy and OA progression. Musculoskeletal-related conditions (e.g., rheumatoid arthritis and disorders of bone density) directly impact joint health and are potential confounders. Medication use (COX-2 inhibitors, corticosteroids) may reflect disease severity or alter joint outcomes. Inclusion of these variables helps minimize selection bias and strengthens the validity of comparative analyses between cohorts. In this study, the distribution between the two compared cohorts needed to be highly similar, achieved through propensity score matching. The matching considered data from the year prior to the index date.

### 2.4. Outcomes Measurement

This study investigated the risk of hip OA patients undergoing THA. The execution of THA was monitored from the day after the index date for five years. All outcomes were delineated using CPT code 27130.

### 2.5. Subgroup Analysis

In subgroup analysis, age statistics were separated for those younger than 60 years old, between 60 and 69 years old, between 70 and 79 years old, and those older than 79 years old. Sex was divided into male and female. Moreover, ethnic background was separated into White Americans, Black or African Americans, and others. For a subgroup with poorer bone density, further research on postmenopausal women, who were defined as females on or above 55, was conducted. In the subgroups related to specific diseases, those diagnosed with obesity, hypertension, RA, and gout were separated from those not diagnosed. For subgroup analyses, each model was constructed based on a restricted cohort defined by specific comorbidities or clinical characteristics. Propensity score matching was then independently performed within the entire database to identify matched controls for each subgroup, rather than selecting from the original control cohort. This approach aimed to reduce selection bias and ensure internal validity within each subgroup comparison.

### 2.6. Time-Variant Hazards and Sensitivity Test

Different time intervals were evaluated to understand the hazard ratio (HR) in this study. The time splits were within one year, within three years, and one to three years, comparing the rates of THA between the two cohorts. To address robustness, we designed three additional models with varying sets of covariates for propensity matching, including both general comorbidities and arthritis-specific factors. Four models were established. Model 1 presented the statistical results between the two groups without PSM. Model 2 involved PSM for age, sex, and ethnic background with relevant chronic diseases, including overweight and obesity, HTN, DM, HF, moderate to severe CKD, dyslipidemia, hospitalization records, and blood pressure. Model 3 involved PSM for age, gender, and ethnic background with arthritis-related diseases, including RA, SLE, AS, psoriasis, gout, HIV, disorders of bone density and structure, and unspecified abnormalities of gait and mobility. Finally, Model 4 is the same as the original analysis.

### 2.7. Statistical Analysis

Matching was performed using 1:1 nearest-neighbor matching without replacement, with a caliper of 0.1 of the standard deviation of the logit of the propensity score. Balance was assessed using SMD < 0.1, which was as acceptable [21]. The Kaplan–Meier approach was utilized to illustrate the risk of undergoing THA. A comparison was conducted between the matched cohorts using the log-rank test. Using the Cox proportional hazard model, the 3-year risk trajectories were depicted through time-varying HRs. The hazard ratios were calculated using Cox proportional hazards models with time-varying covariates, as implemented in TriNetX’s default setting, with balance diagnostics checked via SMD, while Kaplan–Meier curves and forest plots were generated using R4.4.1.

## 3. Results

### 3.1. Baseline Characteristics

The TriNetX US Collaborative Network encompassed 1,504,839 patients with hip OA, of whom 775,910 were diagnosed between 1 January 2016 and 31 December 2020. Excluding those with previous records of undergoing THA or revision of THA and the patients with congenital deformities of the hip and osteonecrosis, the focused population consisted of 752,941 patients. Finally, 23,580 were within the BB cohort, 15,773 within the selective BB cohort, 2564 within the non-selective BB cohort, 8253 within the aBB cohort, and 289,850 within the non-BB cohort. Following the 1:1 propensity score matching between with- and without-BB cohorts, 23,096 patients remained (Figure 1). Before PSM, the average ages of the BB cohort and non-BB cohort were 69 ± 11.9 and 63.3 ± 11.4, with a majority being female (52.4% vs. 56%) and White (72.8% vs. 68.1%). The most prevalent comorbidity in both cohorts was hypertensive disease (17,690 in the BB cohort and 96,056 in the non-BB cohort), followed by dyslipidemia (13,260 vs. 92,257). The distribution between two cohorts is highly similar through propensity score matching, and baseline characteristics of the two cohorts before and after matching are presented in Supplementary Table S1.

### 3.2. Outcomes

**Figure 2:**
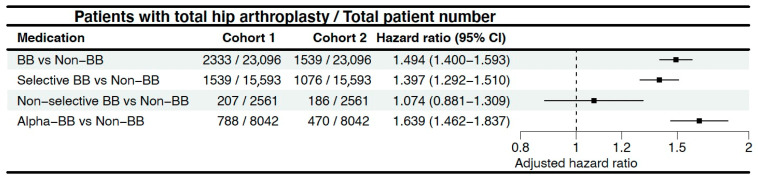
Forest plot showing hazard ratios and 95% confidence intervals for total hip arthroplasty across beta-blocker subtypes compared to the non-BB group.

**Figure 3:**
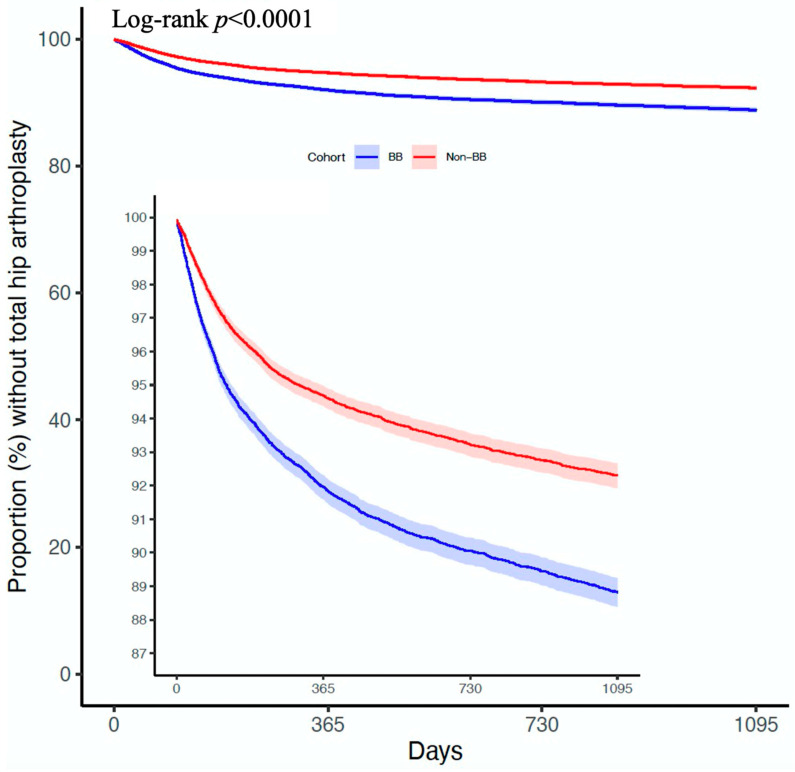
The Kaplan–Meier curve illustrating the cumulative risk of THA in patients with and without beta-blocker use. Censoring events are indicated by tick marks. The number at risk at each time interval could not be displayed due to limitations in the TriNetX platform, which does not support time-specific survival data export.

**Figure 4:**
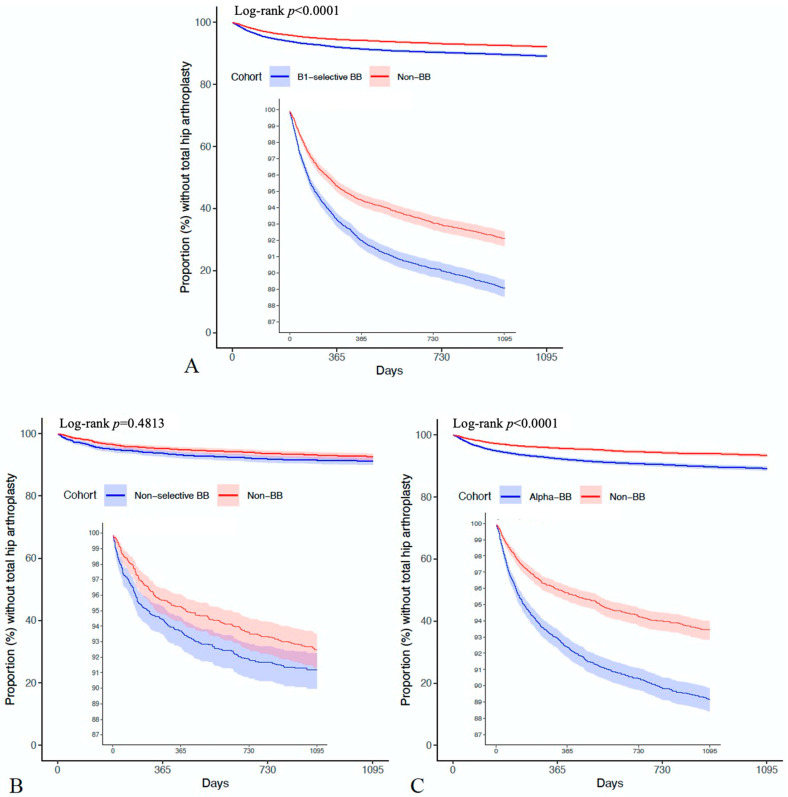
KM curve. (**A**). Cumulative risk of THA after diagnosis of hip OA in patients using selective beta-blockers and those not using beta-blockers. (**B**). Cumulative risk of THA after diagnosis of hip OA in patients using non-selective beta-blockers and those not using beta-blockers. (**C**). Cumulative risk of THA after diagnosis of hip OA in patients using alpha-beta-blockers and those not using beta-blockers.

During the three-year follow-up period from the index day, 2333/23,096 patients in the BB cohort underwent THA, compared to 1539/23,096 cases in the non-BB cohort (HR = 1.494; 95% C.I. = 1.4–1.593) (Figure 2). The cumulative probability curves illustrating THA occurrence in the two cohorts are depicted in Figure 3. Similarly, a significantly higher hazard was observed when comparing the selective BB cohort and aBB cohort to the non-BB cohort. However, compared to the non-BB cohort, the non-selective BB cohort exhibited non-significant different hazards, with 207/2561 vs. 186/2561 (HR = 1.074; 95% C.I. = 0.881–1.309) patients undergoing THA (Figure 2) (Figure 4) (Supplementary Table S3).

### 3.3. Subgroup Analysis, Time-Variant Hazards, and Sensitivity Test

**Figure 5:**
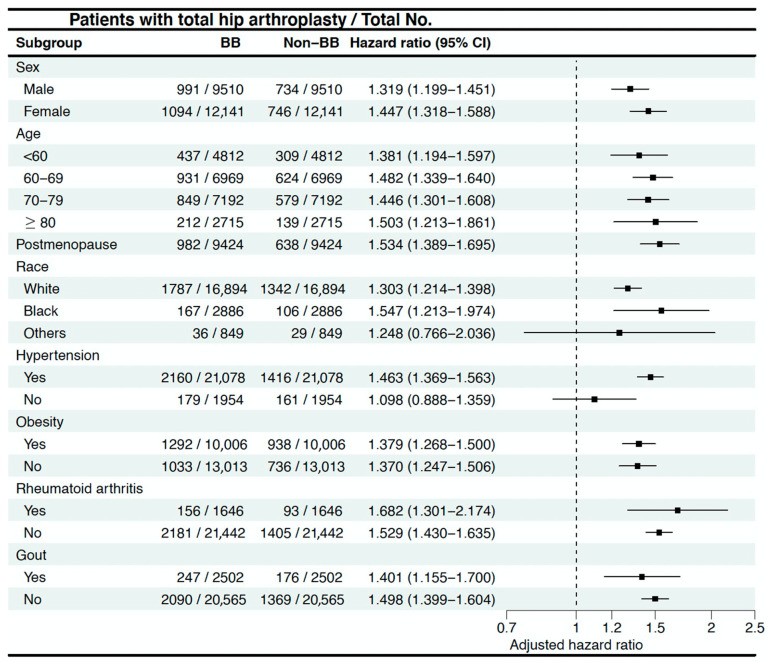
Subgroup analysis.

Subgroup analysis revealed that females had a higher hazard of undergoing THA than males. This may reflect sex-specific variations in joint structure, hormonal influence on cartilage degeneration, or differing thresholds for opting into surgery. The highest HR was observed in the 80-year-old and above age group (212 vs. 239, HR = 1.503; 95% C.I. = 1.213–1.861). Among the included ethnicities, only the population other than White Americans and Black or African Americans showed no statistically significant differences (36 vs. 29, HR = 1.248; 95% C.I. = 0.766–2.036). In terms of related diseases, the impact of BBs was consistently more pronounced in patients with the abovementioned diseases than in those without, except for the cases with gout diagnosis (247 vs. 176, HR = 1.401; 95% C.I. = 1.155–1.7 in with gout subgroup; 2090 vs. 1369, HR = 1.498; 95% C.I. = 1.399–1.604 in without gout subgroup) (Figure 5) (Supplementary Table S4).

Although the cumulative survival curve suggests a decreasing number of individuals undergoing THA from the time of diagnosis, the results of time-varying hazards indicate that the use of BBs is consistently associated with higher THA hazards at any given period, even in the period of 1–3 years (583 vs. 419, HR = 1.369; 95% C.I. = 1.207–1.552). The sensitivity test results show that the highest HR was shown in Model 2 (2356 vs. 1391, HR = 1.669; 95% C.I. = 1.562–1.783) (Supplementary Table S5) (Supplementary Figure S1).

## 4. Discussion

The results of this study, which involved over 47,000 participants after propensity score matching, indicate that patients who took BBs both before and after the diagnosis of hip OA have a higher risk of undergoing THA compared to those who never used BBs. Among them, the impact of aBBs is particularly significant.

In 2019, Georgina et al. planned to investigate the relationship between BB usage and hip and knee OA in the UK through the Clinical Practice Research Datalink. The planned study aimed not only to examine the incidence of OA but also to further explore the pain levels and joint replacement risks in OA patients [22]. By 2021, the results revealed that considering only the period of BB usage, patients using BBs had a lower risk of knee OA compared to the non-user group (aHR = 0.90, 95% CI = 0.83–0.98). However, this association was not found in hip OA (aHR = 0.94, 95% CI = 0.83–1.07). Furthermore, when the results of continuous follow-up after discontinuation of medication were included in the calculations, BB usage was not associated with the risk of OA, and the related pain was higher (Knee: aHR = 1.03, 95% CI = 1.01–1.05; Hip: aHR = 1.04; 95% CI = 1.02–1.07). However, due to the relatively short mean follow-up, the study ultimately did not explore the association between BB use and total joint replacement [8]. Our study, which included more patients and had a longer follow-up duration, found an association between BB usage and a higher likelihood of requiring THA. Another UK study utilized data from the Genetics of Osteoarthritis and Lifestyle (GOAL) study. Its results indicated that BBs were associated with less joint pain (aOR = 0.70; 95% CI = 0.52–0.93) and a lower rate of prescription of analgesics (aOR = 0.74; 95% CI = 0.56–0.97). Moreover, this association remained significant when examining different joints separately, while alpha-blockers showed no significant correlation. The correlation between BBs and joint pain was also influenced by the duration of usage (OR per 1-year increase = 0.96; 95% CI 0.93–0.99) [9]. Overall, in these two studies, the impact of BBs on OA risk, OA-induced pain, and the need for analgesics varied across different joints, modes of drug use, and study methodologies. Now, our study complements previous research by addressing the missing outcome, total joint replacement, and providing a more detailed exploration of the situations when different adrenergic receptors are blocked. In addition, this study includes a more comprehensive analysis based on different BB categories and a more robust PSM. These findings can offer some insights into research in this area.

In previous research on the relationship between the sympathetic nervous system (SNS) and joint health, several experimental models have highlighted its impact on bone and cartilage metabolism. For example, sympathectomy in mice was associated with increased osteoblast activity and bone volume, along with altered expression of adrenergic receptors and osteophyte formation in joint tissues [23]. In rat models, beta-1 adrenergic agonists promoted osteoblast function during mechanical unloading [24], while combinations of α-adrenergic antagonists and β2-agonists reduced cartilage damage and abnormal bone growth [25]. However, some studies reported that BB delayed arthritis onset and severity, though alpha-blockers showed a limited effect [26]. In humans, norepinephrine was shown to influence OA chondrocyte inflammation and metabolism in a dose-dependent manner [27]. Other evidence suggests that β2-adrenoceptor deficiency may protect against stress-induced bone loss [28]. In this study, the higher hazard associated with aBB use may be attributable to its broader vascular effects. Unlike selective beta-1 blockers, aBBs also inhibit alpha-adrenergic receptors, which regulate vascular tone and blood flow. This dual blockade may alter subchondral bone perfusion and remodeling dynamics, potentially accelerating joint degeneration. Additionally, the hemodynamic shifts caused by alpha-blockade may impair microcirculation in the hip joint, which could contribute to cartilage damage or bone marrow lesions—both known predictors of THA. Collectively, these studies suggest a crucial role for adrenergic receptors, including α-adrenergic and β-adrenergic receptors, which are components of the sympathetic nervous system, in joint function and pathology.

Although hypertension is prevalent among patients receiving BBs, our study employed multiple propensity score matching strategies that included hypertension as a covariate to mitigate its confounding effects. Notably, in our sensitivity analyses (Model 2), which specifically adjusted for hypertension and related cardiometabolic diseases, the association between BB use and increased THA risk remained significant. Furthermore, in Model 3, where hypertension was not a matching covariate, a similar association was still observed. These findings suggest that the increased risk is not solely attributable to hypertension itself, but may be more directly linked to the pharmacologic effects of BBs.

This study was not able to classify the severity of newly diagnosed OA of the hip, such as the Tonnis grade. However, as a retrospective observational study, our goal was to explore the effects of different medications across specialties based on past established events, aiming to achieve patient-centered care. Therefore, this study limited BB users to those who used the medication both before and after the diagnosis of OA of the hip, rather than focusing on whether to use BBs after the diagnosis of OA of the hip. This study hopes to pave the way for further research in this field, rather than presenting a conclusive end. The result did not provide recommendations on the choice of antihypertensive medication for patients with OA of the hip. Instead, it aimed to show that when physicians encounter OA of the hip patients who use BBs, they can be better prepared for the possibility of THA. Especially after excluding the first year with a high incidence rate, the second to third years of follow-up in this study still showed significant differences.

Despite extensive data, repeated validations, and cross-comparisons, this study still has some limitations. First, as a limitation inherent to the data source, we were unable to analyze dose–response relationships. The study is unable to assess medication dosage, frequency, or adherence, which limits the precision of exposure classification. Patients who had BB prescriptions may not have taken them consistently, and we could not verify medication compliance. Second, in the classification of BB subgroups, this study did not exclude patients who may have had prior or concurrent exposure to other BB classes. While this may introduce some exposure overlap between subgroups, it reflects real-world prescription patterns and allows for broader generalizability. Third, individuals prescribed BBs may represent a population with a higher cardiovascular comorbidity burden, potentially predisposing them to earlier surgical intervention due to systemic frailty or activity limitation. While we attempted to mitigate this bias via propensity score matching, residual confounding from unmeasured variables (e.g., physical activity, pain threshold, socioeconomic status) remains a possibility. Moreover, patients prescribed aBB may have more severe underlying cardiovascular conditions, which could independently influence the likelihood of undergoing THA. This potential confounding by indication cannot be fully excluded despite adjustment for comorbidities. While residual confounding remains a limitation, the potential direction and magnitude of such bias can be reasonably inferred. For instance, survivor bias may lead to underestimation of arthroplasty risk in patients with greater comorbidity burden—such as those on α–β blockers—who may die before surgery, thereby attenuating observed HRs. Fourth, THA may not be the sole indicator of hip OA prognosis, as the decision to undergo THA depends not only on the severity of hip OA. Future studies could investigate the use of BBs in hip OA patients concerning pain, functional scores, and structural assessments. Fifth, multiple subgroup analyses were conducted without adjustment for multiplicity; thus, these findings should be interpreted cautiously and considered exploratory. Sixth, the BB subclasses were uneven in size, which may affect the precision and comparability of hazard estimates across groups. Additionally, since the dataset does not capture mortality, patients who may have died before undergoing THA were not accounted for, introducing the potential for survivor bias. Finally, THA is not the endpoint of treatment for hip OA patients, and unfortunately, this study did not track post-THA care and survival rates.

In this large-scale, real-world retrospective analysis, beta-blocker use was associated with a modestly reduced risk of total hip arthroplasty in patients with hip osteoarthritis, generating hypotheses for future prospective studies incorporating imaging, functional outcomes, and biomarker validation to determine their clinical applicability. These findings raise questions about potential associations between BB use and THA risk, but should not inform clinical decision-making. While the potential role of adrenergic modulation in joint degeneration remains to be clarified, it represents an area of interest for future clinical investigation, especially in treatment decision-making for hypertensive or multimorbid elderly patients. Future research should move beyond observational associations and explore the mechanistic underpinnings of this relationship. Prospective trials integrating functional scores, patient-reported pain outcomes, and imaging-based structural assessments (e.g., MRI-based cartilage evaluation or bone perfusion studies) may elucidate the specific pathways by which sympathetic regulation affects joint degeneration. In parallel, preclinical studies on adrenergic receptor signaling in subchondral bone and cartilage could offer pharmacological insights into the differential effects of various BB subtypes.

## Data Availability

The data reviewed was from a secondary analysis and can be found at https://trinetx.com/. accessed on 25 June 2024.

## Supplementary Materials

The following supporting information can be downloaded at https://www.mdpi.com/article/10.3390/life15081326/s1. Table S1. Baseline characteristics of study subjects (before and after PSM matching); Table S2. Electrical codes; Table S3. Outcomes of the primary and secondary analysis; Table S4. Outcomes of the subgroup analysis; Table S5. Outcomes of the time variable analysis and sensitivity test; Supplementary Table S6. Inclusion and Exclusion Criteria; Figure S1. Outcomes of the time variable analysis and sensitivity test.
