# Supplementary material for: Beta-Blocker Use and Risk of Hip Arthroplasty in Osteoarthritis: A Retrospective Electronic Health Record Study
Source: Life (Basel). 2025 Aug 20;15(8):1326. doi: 10.3390/life15081326 (PMC12387304; doi:10.3390/life15081326)
Supplement: Supplementary file 1 [file life-15-01326-s001.zip › life-3699469-supplementary.pdf]

Supplementary

Supplementary Table S1. Baseline characteristics of study subjects (before and after PSM matching).
Supplementary Table S2. Electrical codes.
Supplementary Table S3. Outcomes of the primary and secondary analysis.
Supplementary Table S4. Outcomes of the subgroup analysis.
Supplementary Table S5. Outcomes of the time variable analysis and sensitivity test
Supplementary Figure S1. Outcomes of the time variable analysis and sensitivity test.
Supplementary Table S6. Inclusion and Exclusion Criteria.

Supplementary Table S1. Baseline characteristics of study subjects (before and after PSM matching).

Variables	Before PSM			After PSM		
	Beta-blocker cohort (n=23,580)	Without beta-blocker cohort (n=289,866)	SMD	Beta-blocker cohort (n=23,096)	Without beta-blocker cohort (n=23,096)	SMD
**Age at index**						
Mean ± SD	69.0 ± 11.9	63.3 ± 13.4	0.452	68.8 ± 11.9	69.6 ± 12.0	0.065
**Sex, n (%)**						
Female	12362(52.4)	162333(56)	0.072	12169(52.7)	12411(53.7)	0.021
Male	9719(41.2)	110287(38)	0.065	9464(41)	9579(41.5)	0.01
**Race, n (%)**						
White	17164(72.8)	197434(68.1)	0.103	16793(72.7)	17218(74.6)	0.042
Black or African American	2967(12.6)	32891(11.3)	0.038	2905(12.6)	2868(12.4)	0.005
Asian	356(1.5)	4651(1.6)	0.008	349(1.5)	308(1.3)	0.015
Other Race	438(1.9)	8507(2.9)	0.07	433(1.9)	427(1.8)	0.002
**Medical utilization, n (%)**						
Hospital Inpatient Services	11033(46.8)	52903(18.3)	0.64	10560(45.7)	10380(44.9)	0.016
**Comorbidities, n (%)**						
Hypertensive diseases	17690(75)	96056(33.1)	0.926	17206(74.5)	17416(75.4)	0.021
Dyslipidemia	13260(56.2)	92257(31.8)	0.507	12885(55.8)	13042(56.5)	0.014
Diabetes mellitus	7348(31.2)	37911(13.1)	0.446	7030(30.4)	7128(30.9)	0.009
Overweight and obesity	5795(24.6)	40989(14.1)	0.266	5653(24.5)	5589(24.2)	0.006

Disorders of bone density and structure	4157(17.6)	40612(14)	0.099	4106(17.8)	4130(17.9)	0.003
Heart failure	4137(17.5)	8917(3.1)	0.49	3654(15.8)	3343(14.5)	0.038
Chronic kidney disease, stage 3	2338(9.9)	8198(2.8)	0.293	2143(9.3)	2058(8.9)	0.013
Chronic kidney disease, stage 4	586(2.5)	1564(0.5)	0.16	525(2.3)	472(2)	0.016
Chronic kidney disease, stage 5	209(0.9)	494(0.2)	0.099	189(0.8)	167(0.7)	0.011
End stage renal disease	536(2.3)	1201(0.4)	0.162	463(2)	426(1.8)	0.012
Gout	1437(6.1)	6699(2.3)	0.189	1351(5.8)	1249(5.4)	0.019
Unspecified abnormalities of gait and mobility	998(4.2)	8865(3.1)	0.063	992(4.3)	905(3.9)	0.019
Other rheumatoid arthritis	969(4.1)	7743(2.7)	0.08	946(4.1)	847(3.7)	0.022
Psoriasis	425(1.8)	4562(1.6)	0.018	418(1.8)	399(1.7)	0.006
Rheumatoid arthritis with rheumatoid factor	222(0.9)	2257(0.8)	0.018	222(1)	187(0.8)	0.016
Systemic lupus erythematosus	200(0.8)	1627(0.6)	0.034	195(0.8)	177(0.8)	0.009
Ankylosing spondylitis	101(0.4)	803(0.3)	0.026	100(0.4)	82(0.4)	0.012
Human immunodeficiency virus disease	93(0.4)	1086(0.4)	0.003	91(0.4)	95(0.4)	0.003
**Medications, n (%)**						
Glucocorticoids	13232(56.1)	88777(30.6)	0.532	12770(55.3)	12407(53.7)	0.032
Cox-2 inhibitors	1750(7.4)	11530(4)	0.149	1722(7.5)	1762(7.6)	0.007

Bold font represents a standardized mean difference was more than 0.1.
PSM: Propensity score matching, SMD: Standardized mean difference, SD: Standard deviation, BMI: Body mass index.
[a.] Propensity score matching was performed on age at index, sex, ethnic background, medical utilization, comorbidities, and medication usage.

Supplementary Table S2. Electrical codes.

Items	Coding	Codes
Osteoarthritis of hip	ICD10CM	M16
Congenital deformities of the hip	ICD10CM	Q65
Osteonecrosis	ICD10CM	M87
Hypertensive diseases	ICD10CM	I10-I1A
Dyslipidemia	ICD10CM	E78
Diabetes mellitus	ICD10CM	E08-E13
Overweight and obesity	ICD10CM	E66
Heart failure	ICD10CM	I50
Chronic kidney disease, stage 3	ICD10CM	N18.3
Chronic kidney disease, stage 4	ICD10CM	N18.4
Chronic kidney disease, stage 5	CD10CM	N18.5
End stage renal disease	CD10CM	N18.6
Systemic lupus erythematosus (SLE)	CD10CM	M32
Rheumatoid arthritis (with rheumatoid factor)	CD10CM	M05
Rheumatoid arthritis (other)	CD10CM	M06
Ankylosing spondylitis	CD10CM	M45
Psoriasis	CD10CM	L40
Gout	CD10CM	M10
Human immunodeficiency virus [HIV] disease	CD10CM	B20
Disorders of bone density and structure	CD10CM	M80-M85

Unspecified abnormalities of gait and mobility	CD10CM	R26.9
Beta blocking agents	ATC	C07
Beta blocking agents, selective	ATC	C07AB
Beta blocking agents, non-selective	ATC	C07AA
Alpha and Beta blocking agents	ATC	C07AG
Glucocorticoids	ATC	H02
NSAID	ATC	M01A
Cox-2 inhibitors	ATC	M01AH
Total hip arthroplasty	CPT	27130
Revision of total hip arthroplasty	CPT	27134, 27137, 27138
Conversion of previous hip surgery to total hip arthroplasty	CPT	27132
Hemiarthroplasty, hip	CPT	27125
Age at Index	TNX	AI
Female	TNX	F
Male	TNX	M
White Americans	TNX	2106-3
Black or African American	TNX	2054-5
Asian Americans	TNX	2028-9
Other ethnic background	TNX	2131-1
Inpatient Encounter	TNX IMP	Visit

Supplementary Table S3. Outcomes of the primary and secondary analysis.

		Number of Total Hip Arthroplasty			
		Cohort 1	Cohort 2		
	N/Cohort	Events.	Events.	Hazard ratio (95% C.I.)	Log-Rank P
with B blocker vs without B blocker	23096	2333	1539	1.494 (1.4,1.593)	< 0.0001
selective B blocker vs without B blocker	15593	1539	1076	1.397 (1.292,1.51)	< 0.0001
non-selective B blocker vs without B blocker	2561	207	186	1.074 (0.881,1.309)	0.4813
aB blocker vs without B blocker	8042	788	470	1.639 (1.462,1.837)	< 0.0001

Supplementary Table S4. Outcomes of the subgroup analysis.

		Number of Total Hip Arthroplasty			
		Cohort 1	Cohort 2		
	N/Cohort	Events.	Events.	Hazard ratio (95% C.I.)	Log-Rank P
Gender					
Male	9510	991	734	1.319 (1.199,1.451)	< 0.0001
Female	12141	1094	746	1.447 (1.318,1.588)	< 0.0001
Age					
<60	4812	437	309	1.381 (1.194,1.597)	< 0.0001
60-69	6969	931	624	1.482 (1.339,1.64)	< 0.0001
70-79	7192	849	579	1.446 (1.301,1.608)	< 0.0001
>=80	2715	212	139	1.503 (1.213,1.861)	0.0002
Post menopause	9424	982	638	1.534 (1.389,1.695)	< 0.0001
Race					
White	16894	1787	1342	1.303 (1.214,1.398)	< 0.0001
Black	2886	167	106	1.547 (1.213,1.974)	0.0004
Others	849	36	29	1.248 (0.766,2.036)	0.3731
Hypertension					
Yes	21078	2160	1416	1.463 (1.369,1.563)	< 0.0001

NO	1954	179	161	1.098 (0.888,1.359)	0.3886
Obesity					
Yes	10006	1292	938	1.379 (1.268,1.5)	< 0.0001
NO	13013	1033	736	1.37 (1.247,1.506)	< 0.0001
Rheumatoid arthritis					
Yes	1646	156	93	1.682 (1.301,2.174)	< 0.0001
NO	21442	2181	1405	1.529 (1.43,1.635)	< 0.0001
Gout					
Yes	2502	247	176	1.401 (1.155,1.7)	0.0006
NO	20565	2090	1369	1.498 (1.399,1.604)	< 0.0001

Supplementary Table S5. Outcomes of the time variable analysis, sensitivity test, and restricted drug usage.

		Number of Total Hip Arthroplasty			
		Cohort 1	Cohort 2		
	Cohort 1 n./ Cohort 2 n.	Events.	Events.	Hazard ratio (95% C.I.)	Log-Rank P
1Y	23096/23096	1751	1120	1.541 (1.43,1.661)	< 0.0001
3Y	23096/23096	2333	1539	1.494 (1.4,1.593)	< 0.0001
1-3Y	21346/21976	583	419	1.369 (1.207,1.552)	< 0.0001
Model 1	23580/289866	2356	22427	1.201 (1.151,1.253)	< 0.0001
Model 2	23580/23580	2356	1391	1.669 (1.562,1.783)	< 0.0001
Model 3	23580/23580	2356	1755	1.297 (1.22,1.38)	< 0.0001
Model 4	23096/23096	2333	1539	1.494 (1.4,1.593)	< 0.0001

Model 1 Result of comparing two cohorts before PSM.
Model 2 Result of comparing two cohorts with PSM age, gender, and ethnic background with relevant chronic diseases, including Overweight and obesity, HTN, DM, HF, moderate to severe CKD, dyslipidemia, hospitalization records, and blood pressure.
Model 3 Result of comparing two cohorts with PSM of age, gender, and ethnic background with arthritis-related diseases including RA, SLE, AS, psoriasis, gout, HIV, disorders of bone density and structure , and unspecified abnormalities of gait and mobility.
Model 4 Result of comparing two cohorts with PSM same as the original analysis.

Methodology	Patients with total hip arthroplasty / Total No. BB	Non-BB	Hazard ratio (95% CI)
1Y	1751 / 23096	1120 / 23096	1.541 (1.430−1.661)
3Y	2333 / 23096	1539 / 23096	1.494 (1.400−1.593)
1−3Y	583 / 21346	419 / 21976	1.369 (1.207−1.552)
Model 1	2356 / 23580	22427 / 289866	1.201 (1.151−1.253)
Model 2	2356 / 23580	1391 / 23580	1.669 (1.562−1.783)
Model 3	2356 / 23580	1755 / 23580	1.297 (1.220−1.380)
Model 4	2333 / 23096	1539 / 23096	1.494 (1.400−1.593)

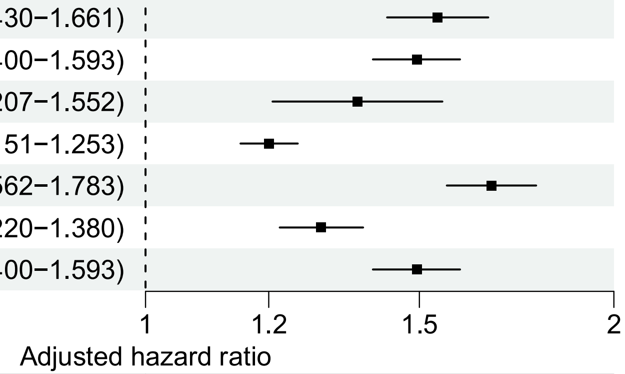

Supplementary Figure S1. Outcomes of the time variable analysis and sensitivity test.

Supplementary Table S6. Inclusion and Exclusion Criteria.

Category	Criteria
Inclusion Criteria	
1. Diagnosis	Patients with hip osteoarthritis (hip OA) diagnosed between January 1, 2016, and December 31, 2020.
2. Data Source	Diagnoses and treatments identified using electronic medical records and coding (details in Supplementary Table 2).
3. BB Cohort Definition	Patients with:<br>• Prescription record for beta-blockers within 1 day to 1 year before index date (hip OA diagnosis)<br>• At least one documented refill after the diagnosis date.
4. Non-BB Cohort Definition	Patients with no prior beta-blocker use at any time.
Exclusion Criteria	
1. Non-Primary OA Conditions	Patients with congenital hip deformities or osteonecrosis, due to different pathophysiological mechanisms and treatment pathways.
2. Prior Surgery	Patients with any record of THA procedures prior to diagnosis of hip OA (to reduce reverse causation and reporting bias).
Additional Notes	
- Dose Limitation	Due to TriNetX dosage granularity, no minimum dose threshold was imposed.
- BB Subgroup Classification	Beta-blocker subtypes were categorized into:<br>• Selective β1-blockers (selective BB)<br>• Non-selective beta-blockers (non-selective BB)<br>• Combined alpha- and beta-blockers (aBB)
